# Mid-term effectiveness, safety, and potential predictors of response of upadacitinib in patients with moderate-to-severe atopic dermatitis: a multicenter observational retrospective study^☆^

**DOI:** 10.1016/j.abd.2024.10.009

**Published:** 2025-04-21

**Authors:** Francisco Javier Melgosa Ramos, Carlos Abril Pérez, Santiago Guillén Climent, María Matellanes Palacios, Juncal Roca Ginés, Javier Sabater Abad, Eduardo Bernia Petit, Andrés Casanova Esquembre, Andrea Estébanez Corrales, Victor González Delgado

**Affiliations:** a Department of Dermatology, Hospital Universitario Doctor Peset, Valencia, Spain; b Department of Dermatology, Hospital Lluís Alcanyís, Xátiva, Valencia, Spain; c Department of Dermatology, Hospital Universitario y Politécnico La Fe, Valencia, Spain; d Department of Dermatology, Hospital Universitario Virgen de los Lirios, Alcoy, Spain; e Department of Dermatology, Hospital Arnau de Vilanova, Valencia, Spain; f Department of Dermatology, Hospital General Universitario, Castellón, Valencia, Spain; g Department of Dermatology, Hospital de la Ribera, Valencia, Spain; h Department of Dermatology, Hospital Frances de Borja, Gandía, Valencia, Spain; i Department of Dermatology, Hospital Clínico Universitario, Valencia, Spain

*Dear Editor,*

The treatment of atopic dermatitis (AD) still presents a therapeutic challenge. The recent approval of targeted therapies has opened new pathways for treatment, with upadacitinib, a selective Janus kinase (JAK) 1 inhibitor,^1^ standing out as a solid candidate. Clinical trials^1^ and real-world studies^2^, ^3^, ^4^, ^5^, ^6^, ^7^ have demonstrated the efficacy and safety of upadacitinib in managing moderate-to-severe AD. These studies highlight its potential to achieve significant improvements in disease severity and patient-reported outcomes.^2^, ^3^, ^4^, ^5^, ^6^, ^7^ However, the variability in patient response requires further investigation into predictors of therapeutic success and the potential impact of early-intervention on long-term disease modification. This study aims to evaluate the mid-term effectiveness and safety of upadacitinib in a real-world setting and identify potential predictors of response in patients with moderate-to-severe AD.

A retrospective non-interventional multicenter study was conducted at the dermatology departments of nine Spanish hospitals from June 2023-to-June 2024. It included adolescents and adults with moderate-to-severe AD treated with upadacitinib 15 or 30 mg in a daily practice setting. No concomitant systemic medication was prescribed. Baseline patient data included age, gender, comorbidities, disease duration, and previous treatments. Disease severity was measured using the Eczema Area and Severity Index (EASI), Body Surface Area (BSA), Investigator Global Assessment (IGA) for AD, and pruritus Numerical Rating Scale (NRS) at Weeks-4, -16, and -24. Quality of life was assessed with the Dermatology Life Quality Index (DLQI). Minimal Disease Activity (MDA)^8^ response (EASI ≤ 3 and peak pruritus NRS ≤ 1) was evaluated at week-24. Drug-related adverse events were recorded throughout the study. The primary endpoint was to analyze the influence of variables (gender, BMI, years of diagnostic delay, presence of atopic comorbidities, cardiovascular risk factors, involvement of special areas, number of previous treatments, and dupilumab exposure) on therapeutic response at week-24. The secondary endpoint was to analyze the evolution of response in terms of absolute EASI, BSA, IGA, DLQI, and pruritus NRS scores at Weeks-4, -16, and -24, and the safety profile of the drug. Descriptive statistics were used to evaluate the characteristics of the sample. The Shapiro-Wilk test was used to assess the normality of the variables. Continuous variables were expressed as mean and Standard Deviation (SD). Qualitative variables were expressed as relative frequency distributions. To explore potential associated factors, multiple linear regression was used, considering the absolute reduction in EASI and NRS as the dependent variables. Statistical significance was considered if p-values were less than 0.05.

Sixty-three patients with a mean age of 42.65-years (SD = 18.42) were included. Patients’ characteristics are detailed in Table 1. Upadacitinib was the first systemic treatment for 16.1% of patients. Most patients (60.5%) received a daily dose of upadacitinib of 30 mg. Only 5.5% of patients switched from 30 mg to 15 mg once achieving optimal response. The mean exposure to upadacitinib 15 or 30 mg was 11.9 months (SD = 8.38). Treatment responses in terms of EASI, IGA, BSA, DLQI, and peak pruritus NRS are detailed in Table 2. No significant differences were observed between patients treated with 15 vs. 30 mg doses and those previously treated with dupilumab vs. not treated. We did not observe differences in the effectiveness of upadacitinib among different phenotypes of AD. MDA^8^ criteria at week-24 were achieved by 55.5% (95% CI 42.72‒67.28) of patients. Among the variables analyzed, the number of years of diagnostic delay (p = 0.013) and the number of previous treatments (p = 0.038) showed a statistically significant association with EASI response, and the number of previous treatments (p = 0.010) showed a statistically significant association with pruritus NRS at week-24, respectively. The presence of a family history of AD was associated with a statistically significant increase in the EASI score (p = 0.036) (Table 3 and Table 4). Twenty patients were followed up until week-52 and presented a mean absolute EASI of 2.57 (SD = 5.21), 90% of IGA 0‒1, and NRS pruritus of 0.89 (SD = 1.94). 75% (95% CI 64.31‒85.69) of them achieved MDA^8^ defined by EASI ≤ 3 and NRS pruritus ≤ 1. The safety profile of upadacitinib was favorable. Infections or Major Adverse Cardiovascular Events (MACEs) were not reported. Five patients experienced transient and isolated lymphopenia (750–1000 mm^3^), which resolved spontaneously. During the follow-up, no patient discontinued the drug.

**Table 1:** Baseline characteristics of the patients.

n = 63	
**Age (years) (SD)**	42.65 years (18.42)
**Gender (Female)**	43.64%
**Family history of AD**	29.27%
**BMI (Kg/m^2^) (SD)**	24.11 (8.05)
**Years of diagnostic delay (SD)**	4.56 (6.16)
**Atopic comorbidities**	69.09%
Food allergy	25.93%
Rhinitis	50.91%
Asthma	41.82%
**Cardiovascular risk factors**	30.91%
**Special areas**	85.45%
Hands eczema	56.36%
Facial/Neck	74.55%
Genital	20.00%
**Basal EASI (SD)**	18.98 (7.25)
**Basal Peak pruritus NRS (SD)**	7.60 (1.61)
**Basal IGA (SD)**	3.10 (0.62)
**Number of previous treatments (SD)**	1.35 (0.95)
**Previous classical treatments or phototherapy**	
Cyclosporine	43.64%
Methotrexate	9.09%
Azathioprine	9.09%
Mycophenolate	3.29%
Phototherapy	12.73%
**Previous advanced treatments**	
Dupilumab	44.3%
Tralokinumab	3.64%
Baricitinib	1.82%
Abrocitinib	1.82%
**Time of exposure (months) to upadacitinib (SD)**	11.92 (8.38)

BMI: Body Mass Index; EASI: Eczema Area and Severity Index; NRS: Numerical Rating Scale; IGA: Investigator Global Assessment.

**Table 2:** Treatment response to upadacitinib during the follow-up period.

	Basal Mean^a^ (SD)	Week 4 Mean (SD)	Week 16 Mean (SD)	Week 24 Mean (SD)
EASI (SD)	18.98 (7.25)	3.02 (3.81)	1.15 (2.22)	0.84 (2.27)
BSA (SD)	24.66 (15.78)	3.94 (4.77)	1.84 (3.96)	0.85 (2.74)
DLQI (SD)	16.48 (6.42)	3.30 (5.27)	1.48 (2.64)	0.77 (1.92)
IGA (Value and %)	3‒4 (87.5%)	3‒4 (4.8 %)	3‒4 (4.5%)	3‒4 (2.5%)
	2 (12.5%)	2 (12.2%)	2 (8.5%)	2 (5.5%)
		0‒1 (83%)	0‒1 (87%)	0‒1 (93%)
Peak pruritus NRS (SD)	7.60 (1.61)	1.61 (1.87)	0.70 (1.30)	0.45 (1.25)

EASI, Eczema Area and Severity Index; BSA, Body Surface Area; DLQI, Dermatology Life Quality Index; IGA, Investigator Global Assessment; NRS pruritus, Numeric Rating Scale for pruritus.

a The mean and standard deviation were used, except for IGA.

**Table 3:** Multiple linear regression model exploring the influence of variables (gender, BMI, years of diagnostic delay, presence of atopic comorbidities, cardiovascular risk factors, involvement of special areas, number of previous treatments, and dupilumab exposure) on therapeutic response in terms of EASI at week 24.

EASI/Variables	Coef	Std Err	T	p>|*t*|	[0.025	0.975]
Intercept	21.4782	4.598	4.674	0.0	12.384	30.572
Gender	−1.6871	1.211	−1.393	0.165	−4.078	0.704
BMI (Kg/m^2^)	−0.0332	0.296	−0.112	0.911	−0.616	0.55
Years of diagnostic delay	0.3592	0.142	2.528	0.013	0.078	0.64
Family history	2.7874	1.322	2.108	0.036	0.175	5.4
Atopic comorbidities	−1.1373	1.225	−0.928	0.355	−3.554	1.279
CVD risk factors	2.7548	1.557	1.769	0.09	−0.409	5.718
Special areas	1.4782	1.598	0.925	0.357	−1.679	4.635
Number of previous treatments	0.9873	0.472	2.092	0.038	0.053	1.921
Dupilumab	−0.1421	0.296	−0.48	0.632	−0.724	0.439

BMI, Body Mass Index, CVD, Cardiovascular Disease.

**Table 4:** Multiple linear regression model exploring the influence of variables (gender, BMI, years of diagnostic delay, presence of atopic comorbidities, cardiovascular risk factors, involvement of special areas, number of previous treatments, and dupilumab exposure) on therapeutic response in terms of pruritus NRS at week 24.

Pruritus NRS/Variables	Coef	Std Err	*t*	p>|*t*|	[0.025	0.975]
Intercept	4.4782	0.598	7.491	0.0	3.298	5.659
Gender	−0.6871	0.455	−1.51	0.133	−1.587	0.213
BMI (Kg/m^2^)	0.0332	0.116	0.287	0.774	−0.195	0.261
Years of diagnostic delay	0.0592	0.056	1.056	0.293	−0.051	0.169
Family history	0.4874	0.518	0.94	0.349	−0.534	1.509
Atopic comorbidities	−0.6373	0.482	−1.322	0.188	−1.588	0.313
CVD risk factors	0.7548	0.626	1.206	0.23	−0.482	1.991
Special areas	0.4782	0.618	0.774	0.44	−0.742	1.698
Number of previous treatments	0.4873	0.186	2.62	0.01	0.122	0.853
Dupilumab	0.0421	0.116	0.362	0.717	−0.187	0.271

BMI, Body Mass Index, CVD, Cardiovascular Disease.

In our series, treatment with upadacitinib in patients with moderate-to-severe AD showed significant mid- and long-term efficacy. These results are similar to those already reported in clinical trials and major real-world series.^1^, ^2^, ^3^, ^4^, ^5^, ^6^, ^7^, ^8^ Our patients exhibited severe disease profiles and resistance to multiple systemic therapies, including biologics, which contrasts with the profiles typically seen in clinical trials.^1^ Additionally, by week-16, more than 50% of patients achieved MDA (defined in our series as EASI ≤ 3 and peak pruritus NRS ≤ 1), a more stringent response criterion recently proposed by Silverberg JI et al.,^8^ which encompasses clinical response and Patient-Reported Outcomes (PROs). The MDA percentage increased to 75% by week-52. We wanted to highlight that despite the high proportion of patients achieving substantial improvements in EASI, peak pruritus NRS, and IGA index, the number of years of diagnostic delay and the number of previous treatments could negatively influence therapeutic response, highlighting the importance of early treatment.^9^ This reinforces the recent hypothesis surrounding advanced and long-standing cases, that the main cytokines involved in cutaneous inflammation, particularly Th2-cytokines (Interleukin [IL]-4, IL-5, IL-13, IL-31)/ JAK-1 pathway may also be implicated in promoting itch at the neuronal level (neuroinflammation), thus chronicling the process.^10^ This raises the question of whether early intervention for AD could lead to a modification of the disease, either in severity or in future persistence.^9^ This study has some limitations, with the main one being derived from its observational and retrospective nature, which does not allow missing data to be retrieved. Additionally, only patients who used upadacitinib for at least 24-weeks were analyzed, and those who discontinued the drug were not assessed. This may overestimate the rates of success and safety in the current sample other limitations include the small sample size and the statistical adjustments performed, which may impact the accuracy and generalizability of the results. In conclusion, upadacitinib is presented as an effective alternative in the treatment of moderate-to-severe atopic dermatitis, with significant improvements observed in both mid- and long-term follow-up. Despite the promising results, early intervention remains crucial due to the potential negative impact of diagnostic delays and multiple previous treatments on therapeutic response.

## Authors’ contributions

Francisco Javier Melgosa Ramos: Contributed to the conception, design, data collection, interpretation, writing-draft preparation and review of the present work.

Carlos Abril Pérez: Contributed to the conception, data collection, interpretation and review of the present work.

Santiago Guillén Climent: Contributed to data collection, interpretation and review of the present work.

María Matellanes Palacios: Contributed to data collection, interpretation and review of the present work.

Juncal Roca Ginés: Contributed to data collection, interpretation and review of the present work.

Javier Sabater Abad: Contributed to data collection, interpretation and review of the present work.

Eduardo Bernia Petit: Contributed to data collection, interpretation and review of the present work.

Andrés Casanova Esquembre: Contributed to data collection, interpretation and review of the present work.

Andrea Estébanez Corrales: Contributed to data collection, interpretation and review of the present work.

Victor González Delgado: Contributed to the conception, design, data collection, interpretation, writing-draft preparation and review of the present work.

## Financial support

None declared.

## Conflicts of interest

None declared.
